# Differentiation of European yellow rust subraces within the ‘Warrior(-)’ genetic group

**DOI:** 10.1371/journal.pone.0323046

**Published:** 2025-05-23

**Authors:** Claudia Breitkreuz, Philipp Schulz, Anne-Kristin Schmitt, Kerstin Flath, Tiphaine Vidal, Amelia Hubbard, Kostya Kanyuka, Andreas Stahl, Jens Keilwagen, Dragan Perovic

**Affiliations:** 1 Julius Kühn-Institut (JKI), Federal Research Centre for Cultivated Plants, Institute for Resistance Research and Stress Tolerance, Quedlinburg, Germany; 2 Julius Kühn-Institut (JKI), Federal Research Centre for Cultivated Plants, Institute for Plant Protection in Field Crops and Grassland, Kleinmachnow, Germany; 3 Université Paris-Saclay, INRAE, Palaiseau, France; 4 Niab, Park Farm, Histon, Cambridge, United Kingdom; 5 Julius Kühn-Institut (JKI), Federal Research Centre for Cultivated Plants, Institute for Biosafety in Plant Biotechnology, Quedlinburg, Germany; 6 Julius Kühn-Institut (JKI), Federal Research Centre for Cultivated Plants, Institute for Resistance Research and Stress Tolerance, Kleinmachnow, Germany; Borlaug Institute for South Asia-CIMMYT, INDIA

## Abstract

Wheat yellow rust is one of the most destructive and rapidly evolving wheat diseases worldwide, particularly in Europe. In 2011, the previously clonal European yellow rust races were replaced by a presumably sexually derived population, characterized as the new race called ‘Warrior’. This race acquired additional virulence, leading to the emergence of ‘Warrior(-)’ in 2013. Since 2017, Warrior(-) has undergone further diversification into subraces, named after the wheat cultivars on which they were first detected: ‘Amboise’, ‘Benchmark’ and ‘Kalmar’. While none of these subraces have been directly linked to the breakdown of a specific resistance gene, they exhibit distinct infection patterns on wheat differential sets. The lack of genetic resolution required to develop reliable genetic markers for diagnosis purposes is addressed in this study. Yellow rust isolates from the ‘Warrior(-)’ race group were collected as part of monitoring initiatives in France, Germany, Austria, and the UK. Marker development was based on a training set of German and French isolates with known pathotypes collected between 2017 and 2021. Using genotyping-by-sequencing (GBS) and whole genome sequencing (WGS), comparisons of subraces with Fisher’s exact test (case-control study) identified 14 significant single nucleotide polymorphisms (SNPs). From these, we established four functional genetic markers capable of distinguishing between the ‘Amboise’ and ‘Benchmark’ subraces, though differentiation of ‘Kalmar’ was not successful. These four markers were validated on two independent control groups of isolates sampled in 2021 and 2022 from the UK (*n* = 30) and Germany (*n* = 40), respectively. While subrace predictions were accurate for the German group, predictions for the UK group failed. Principal Coordinates Analysis (PCoA) of genetic distances revealed a strong origin-driven effect, further confirmed by coverage analysis of the GBS data, which demonstrated an impact on the frequency and distribution of cleavage sites. Thus, this study provides a valuable tool for future yellow rust monitoring efforts while also highlighting significant origin-dependent effects that must be considered in genetic analyses.

## Introduction

Yellow rust (or stripe rust), caused by *Puccinia striiformis* f. sp. *tritici* (*Pst*), is characterized by the appearance of yellow pustules arranged in stripes on wheat leaves [1]. As a member of Basidiomycota, yellow rust has a complex life cycle, including both asexual reproduction on wheat and sexual reproduction on its alternate host, *Berberis vulgaris* [2]. Epidemics of this obligate biotrophic fungus lead to significant global yield losses in wheat production areas [3]. While it has traditionally been considered a disease of cooler climates, such as northern Europe and the Pacific Northwest of the USA, recent epidemics in warmer regions including South Asia and northern Africa, suggest an adaptation to new environmental conditions [4].

The pathogen is believed to have originated in the Himalayan region, where sexual recombination contributes to highly diverse populations with extensive genetic and pathogenic variation [5]. Through successive field-to-field spread, facilitated by its polycyclic asexual reproduction during each growing season [6], or via long-distance wind dispersal of airborne spores [7–8], yellow rust has spread to Europe, North America, and Australia over the last century [5,9]. In these regions, clonal populations have become established [5,10,11], demonstrating the pathogen’s strong adaptive capabilities. Adaptation occurs either through stepwise evolution [10] or somatic exchange events [12], allowing the pathogen to evade recognition by host disease resistance (*R*) genes.

In Europe, comprehensive monitoring efforts have documented the gradual evolution of yellow rust races with virulence against various *R* genes since the 1930s [13]. In addition to the traditional classification into physiological races (or pathotypes), advancements in genetic analysis [14–16] have enabled the classification of yellow rust isolates into distinct genetic groups [5,17]. These genetic groups, designated as ‘Pst’ followed by a number, have been found to strongly correlate with pathotypes [18]. A study comparing past (1958–1991) and more recent (1992–2009) yellow rust populations on a global scale revealed significant genetic differences between isolates from northwestern (NW) Europe and those from South Asia and Africa. Moreover, NW European populations exhibited the lowest genetic diversity and highest stability over time [17]. These clonal populations have been classified as the NW European genetic group, belonging to the PstS0 genetic group, which includes the races ‘Brigadiera’, ‘Solstice_oakley’ and ‘Tulsa’ [7].

A major shift in the European yellow rust population structure occurred in 2011 with the emergence of two new genetic groups, termed ‘Warrior’ (PstS7) and ‘Kranich’ (PstS8). This divided the population into the pre-2011 clonal lineages and the newly introduced, possibly sexually-derived, lineages of ‘exotic’ origin [7,19]. The exotic groups show strong genetic relatedness to isolates from the Himalayan region and carry unique alleles previously absent in European clonal populations [5]. Both ‘Kranich’ and ‘Warrior’ spread rapidly across Europe and were highly abundant even in their first year of detection, replacing races belonging to the genetic groups PstS0, PstS2, PstS3, ‘Triticale aggressive’ and ‘Tulsa’ in Denmark, France, Germany, Spain, Sweden, and the UK [18]. Their rapid spread was facilitated by the fact that widely grown commercial wheat varieties carried durable resistance to the previously dominant pathogen populations but lacked resistance to the newly introduced ones [20].

Since their introduction, the ‘Warrior’ race has increased in prevalence and evolved additional virulence against previously resistant cultivars [7]. Today, the resulting ‘Warrior(-)’ (PstS10) group dominates the European yellow rust population. Within this group, further differentiation has led to the emergence of several subraces, named after cultivars (cvs.) on which they were first detected and which were previously considered resistant, including cvs. Amboise, Benchmark and Kalmar. The yellow rust resistance gene complement within these cultivars remains unknown, and their susceptibility to specific pathogen subraces cannot yet be linked to known *R* genes [18]. Due to the relatively recent establishment of these subraces within PstS10 in Europe, studies on their genetic background remain limited, particularly regarding their emergence mechanisms and distinct virulence profiles. Identifying pathotypes (or virulence profiles) currently relies on labor-intensive and time-consuming bioassays using wheat differential sets. Therefore, the development of a marker-based approach to distinguish between the ‘Amboise’, ‘Benchmark’ and ‘Kalmar’ subraces would be of significant interest for diagnostic purposes.

We applied genotyping-by-sequencing (GBS) to a set of ‘Warrior(-)’ isolates with known subrace identities, collected from four European countries between 2017 and 2022. Using a case-control-study approach, we correlated GBS data with the fungal subraces ‘Amboise’, ‘Benchmark’ and ‘Kalmar’ to identify single nucleotide polymorphisms (SNPs) and insertion/deletions (InDels) associated with their phenotypes. Based on these findings, we developed molecular markers for subrace identification. In addition to the marker-based approach, we analyzed genetic variation among isolates from different geographical regions, revealing location-dependent effects that may indicate further shifts in European yellow rust populations. Overall, this study provides valuable tools for monitoring the spread and prevalence of ‘Warrior(-)’ subraces across Europe.

## Materials and methods

### Origin, genotyping and phenotyping of yellow rust isolates

As part of national and international monitoring programs, wheat leaf samples infected with yellow rust were collected by farmers or local contributors from fields and send to reference centers for pathotyping. In this study, samples originated from Germany, Austria and France collected between 2017 and 2022 (Global Rust Reference Center (GRRC); RustWatch [18]), as well as from the UK, collected in 2021 (United Kingdom Cereal Pathogen Virulence Survey, UKCPVS, [21]) (Table S1). Pathotyping of yellow rust isolates were performed at the respective reference centers.

As this study focused exclusively on isolates belonging to the genetic group PstS10 (‘Warrior(-)’), yellow rust isolates were pre-selected based on single sequence repeat (SSR) marker analysis. Total fungal DNA was extracted from urediniospores obtained from a single wheat leaf heavily infected with yellow rust using the Qiagen Plant DNeasy kit, according to the manufacturer’s protocol. Multiplex PCR was performed using the Qiagen Type-it microsatellite kit according to the manufacturer’s instructions, with slight modifications as defined in Thach et al. [17]. Sample analysis was conducted at KiGene on the ABI-platform (Karolinska Institute, Stockholm, Sweden), and band sizes were determined using Osiris [22]. Isolates confirmed to match the PstS10 profile were subsequently multiplied for pathotyping.

Pathotyping and subrace designation were carried out using seedling bioassays following the method of Rahmatov et al. [23], with a modification to the carrier substrate: 0.1% agar was used instead of mineral oil for plant inoculation with spore suspensions. Infection capability was tested on the standard differential cultivar set with the additional cultivars cvs. Kalmar or Nemo, Benchmark and Amboise. A key characteristic of the subraces is that ‘Amboise’ is virulent on all three additional cultivars, whereas ‘Benchmark’ is only virulent on cv. Benchmark, and ‘Kalmar’ only on cvs. Nemo or Kalmar (Table S2).

In total, the study included 192 isolates belonging to the Warrior(-) genetic group, divided into a marker development group (training set) and two control groups (test sets). The training set consisted of German and French isolates collected between 2017 and 2021 (*n* = 122 isolates), whereby pathotype classification could not be determined for all isolates (49 ‘Amboise’, 25 ‘Benchmark’, 37 ‘Kalmar’ and 11 unclassified isolates). The first test set included German isolates collected in 2022 (testG: *n* = 40 isolates), which had been pathotyped but not subjected to GBS. The second test set consisted of UK isolates that had undergone GBS analysis, with pathotypes predicted based on genetic markers (testUK: *n* = 30 isolates) and subsequently compared with pathotyping results to assess accuracy.

### DNA extraction from wheat leaf material and fungal spores

For GBS analysis, fungal DNA was extracted from 0.02 mg of dried urediniospores using a modified cetyl trimethylammonium bromide (CTAB) method based on Murray and Thompson [24]. Briefly, spores were ground with 200 mg of sterile sand and mixed with 1 mL of extraction buffer (50 mM Tris-HCl (pH 8), 150 mM NaCl, 100 mM EDTA (pH 8)). Subsequently, 50 µL of 20% sodium dodecyl sulfate (SDS) was added, and the mixture was gently shaken at room temperature for one hour. To facilitate cell lysis, 150 µL of 5 M NaCl and 130 µL of 10% CTAB/0.7 M NaCl were added, followed by incubation at 65°C for 20 min. DNA was then isolated from the lysed cellular components using one volume of dichlormethan/isoamylalcohol (24:1). The mixture was vortexed and centrifuged at 10.000 rpm for one min, after which the upper aqueous phase containing DNA was transferred to a new tube for further processing. DNA was precipitated by adding 0.7 volumes of ice-cold isopropanol, followed by incubation at room temperature for 10 min. Samples were centrifuged at maximum speed for 20 min, and the resulting DNA pellets were washed twice with 70% ethanol. To remove RNA contamination, the pellets were resuspended in 500 µL of TE buffer (pH 8) containing RNase T1 and incubated at 37°C for one hour. DNA was then precipitated by adding 3 M sodium acetate (NaOAc, pH 5) and 0.7 volumes of isopropanol at a 1:10 ratio, followed by incubation at room temperature for 10 min. The centrifugation and ethanol-washing steps were repeated, and the final DNA pellets were resuspended in 40 µL of TE buffer (pH 8). DNA quantity and quality were assessed using a Qubit Fluoremeter (Invitrogen, Thermo Scientific, Waltham, Massachusetts, USA) and a Nanodrop spectrophotometer (Thermo Scientific, Waltham, Massachusetts, USA).

### Partial genome sequencing using genotyping-by-sequencing (GBS)

#### *In silico* restriction enzyme digest.

An *in silico* approach is the preferred method for evaluating the potential performance of target-oriented systems, such as enzymatic digestion of genomes. It allows for the simultaneous comparison of different restriction enzymes by analyzing the number and distribution of cleavage sites, as well as fragment sizes – a crucial factor for sequencing methods with defined read lengths, such as Illumina (150–600 bps) – without the need for labor-intensive and costly laboratory experiments.

We applied the *in silico* approach to a yellow rust reference genome (GenBank assembly GCA_021901655.1) using the SimRad package [25] with slight modifications (R-script, S1 File). Cleavage sites for two restriction enzymes (site 1: 5’…, site 1: …3’; site 2: 5’…, site 2: …3’) were set as parameters, along with a minimum fragment size of 150 bp and a maximum of 600 bp. The performance of established single and double enzymatic digests was assessed, including *Btg*I and *Bam*HI [26], *Hha*I [27], and *Pst*I and *Msp*I [28]. The optimal fragment distribution was observed for a double digest using *Msp*I (a “rare-cutter”; site 1: 5’…C/ CGG…3’) and *Pst*I (a “common-cutter”; site 2: 5’…CTGCA/ G…3’) [28].

#### GBS library construction and sequencing.

The training and testUK sets were sequenced using the GBS method (n = 152 isolates). The GBS protocol of Wendler et al. [29] was applied with slight modifications. Briefly, fungal genomic DNA concentrations were adjusted to 20 ng/µL and digested overnight at 37°C with the restriction enzymes *Msp*I (NEB #R0106S; New England Biolabs GmbH, Frankfurt, Germany) and *Pst*I (NEB #R3140) (Mastermix details in Table S3). Fragmentation patterns of the genome samples were assessed using electrophoresis in a 2% Ultra PureTM Agarose Gel. DNA fragments between 150 bp and 600 bp were excised purified using the MinElute Gel Extraction Kit (Qiagen) following the manufacturer’s instructions. For Illumina sequencing, hybridization mixtures of reading primers (Table S4, Adaptermix P5.7_GBS) were designed to bind complementary to the *Msp*I and *Pst*I overhangs. After ligation of reading primers (Table S5 and Table S6), the fragments were tagged with unique barcodes (set from Beier et al. [30]), and equipped with P5 and P7 adapters, enabling library fragments to attach to the Illumina flow cell surface (Index-PCR, Table S7). Samples were pooled at equal concentrations of 6 ng per sample. The pooled library’s size and quality were assessed using the Agilent 2100 Bioanalyzer system (Agilent Technologies, Palo Alto, CA, United States). Single-end sequencing was performed using the Illumina MiSeq® Reagent Kit v3 (150 cycle), generating sequences of up to 150 bp in length. Raw sequencing data have been uploaded to the National Center for Biotechnology Information (NCBI) under BioProject PRJNA1214722.

### Whole genome sequencing

A total of 24 isolates (originating from Germany and France), comprising eight from each subrace (Table S1), with unequivocal pathotyping results, were selected for whole genome sequencing using the Illumina MiSeq system (Illumina, San Diego, United States). DNA concentrations were adjusted to 200 ng/µL. Sequencing libraries were prepared using the Illumina DNA Prep Tagmentation Kit and equipped with IDT-ILMN Nextera DNA UD Indices (96 Indices) (Illumina, San Diego, United States) according to manufacturer’s instructions. Paired-end sequencing was performed using the MiSeq Reagent Kit v3 (600-cycle), generating extended read lengths of up to 2 x 300 bp (coverage = 24%). Raw sequencing data have been uploaded to NCBI under BioProject PRJNA1214722.

### Data analysis

#### Variant calling.

Sequencing files were uploaded to the internal JKI Galaxy Server [31] and the workflow presented in Fig S1 was followed. Using Trim Galore! [32], sequencing adapters and low-quality ends (<30%) were removed from reads. Only sequences with a maximum error rate of <0.1 and lengths ≥ 50 bp were retained for mapping against the reference genome of yellow rust (PstS1: BioProject PRJNA757545: assembly at the chromosome level Pst134E36_v1_pri (principal haplotype) and Pst134E16_v1_alt (alternate haplotype)) using the BWA-MEM tool [33]. The number of unmapped reads and coverage analyses were assessed with samtools [34] (Table S1) and bedtools [35], respectively. The results presented in this study correspond to the alternate haplotype (Pst134E16_v1_alt). To identify variants in the sequencing data relative to the reference genome, variant calling was performed using BCF tools in Galaxy [36]. To minimize false variant calls due to sequencing errors, filter options for minimum SNP quality was set to 40, with at least 4 reads covering the position per sample, and the threshold for uncovered samples per variant was set to 30% using VCF filter [37].

For marker development, variant calling was performed for the training set and the resulting variant calling file (vcf) contained all detected variants including single nucleotide polymorphisms and insertion/deletions (training: n = 13,075 SNPs). For genetic distance analysis, training and testUK sets were analyzed collectively (n = 152 isolates). The output included either all detected variants (Dataset 0, Table 1) or a dataset prioritizing SNPs (Dataset 1, Table 1). Subsequently, various filtering steps were applied to control for minor allele frequency (MAF) and heterozygosity (HET) (Datasets 2 and 3, Table 1), resulting in a consecutive reduction in the number of variants (Table 1).

**Table 1:** Summary of sequential filtering steps applied to combined training and testUK sets. The number of variants and genetic distance based on Roger’s Distance (RD) are given for each filtering step and were named Dataset 0-3.

Filtering steps	Dataset	Variants	Genetic Distance, RD
VCF filter (Galaxy, any variants)	0	12,791	–
VCF filter (Galaxy, only SNPs)	1	12,163	0–0.08
HapMap-Filter (Galaxy, MAF > 0.05)	2	3,463	0–0.08
HapMap-Filter (Galaxy, MAF > 0.05 and HET threshold = 0.5)	3	263	0–0.31

#### Genetic distance.

Genetic distances (Rogers’ distance [38]) were calculated in Galaxy for German, French and UK isolates (training and testUK sets: *n* = 152 isolates). Principal coordinate analysis (PCA) of genetic distances and discriminant analysis of principal components (DAPC) were performed and visualized in R [39] (v 4.0.5, R Core Team, 2021] using the stats (v4.0.5), ggplot2 (v3.3.4), and ggspatial (v1.1.6) packages, as well as rnaturalearth (v0.1.0), rnaturalearthdata (v0.1.0) and sf (v1.0-8) for visualizing geographical GBS patterns.

#### Case control study.

To identify significant SNPs in the training set (n = 122 isolates) that differentiate fungal subraces ‘Amboise’ (A), ‘Benchmark’ (B), and ‘Kalmar’ (K), a case control study was conducted using SnpEff & SnpSift toolbox [40]. For this analysis, the software performed Fisher’s exact test on contingency tables under two models: a dominant model (Ref = A/A vs. Alt = A/a and a/a), and a recessive model (Ref = a/a vs. Alt = A/A and A/a). To additionally assess heterosis effects (Ref = A/a vs. A/A and a/a), contingency tables and Fisher’s exact test were calculated in R (v 4.0.5, [39]). A TFAM-file was prepared with the following groupings:

1) K (Case = 2) vs. B (Control = 1), A unknown (=0)
2) K (=2) vs. A (=1), B unknown (=0)
3) B (=2) vs. A (=1), K unknown (=0)
4) K (=2) vs. A (=1) and B (=1)
5) B (=2) vs. A (=1) and K (=1)
6) A (=2) vs. B (=1) and K (=1)

Isolates without a clear assignment were treated as neutral (=0). Significant SNPs (*p* < 0.05), which allowed differentiation between subraces (e.g., ‘Amboise’ = AA, ‘Benchmark’ = aa, ‘Kalmar’ = Aa), were selected for marker development.

### Marker development

Marker development was based on GBS analysis results from yellow rust isolates of the trainings set (n = 122 isolates). UK isolates from 2021 (testUK set: *n* = 30 isolates) and German isolates from 2022 (testG set: *n* = 40 isolates) served as control groups. Mapping files were extracted from the Galaxy server and loaded into IGV Viewer (v2.16.2, [41]). Within the alignments, significant SNPs identified in the case-control study were examined, and upstream or downstream sequences were extracted for Kompetitive Allele-Specific PCR (KASP) primer design using the online tool BatchPrimer3 [42]. The primer pairs for markers with fluorescence dyes FAM and HEX are listed in Table S8.

KASP reactions were performed on the Analytic Jena qTOWER^3^G platform in a 96-well format. Reactions were set up in 10 µl volumes using the KASP-TF V4.0 2x Mastermix (BiosearchTechnologies) and run according to manufacturer’s conditions, with three additional ‘Recycling’ cycles. A total of 30 ng DNA was used per reaction. Based on the manufacturer’s troubleshooting guide, the primer mix volume was adjusted to 0.28 µL per 10 µL reaction, instead of the standard 0.14 µL. Since the markers differentiate between homozygous (identical to reference or alternate) and heterozygous states, the FAM/HEX fluorescence ratio was calculated as follows: FAM/HEX > 1, homozygous for the reference allele; FAM/HEX = 1, heterozygous; FAM/HEX < 1, homozygous for the alternate allele.

## Results

### Comparison of *in silico* and wet laboratory conditions for enzyme digests

**Fig 1:**
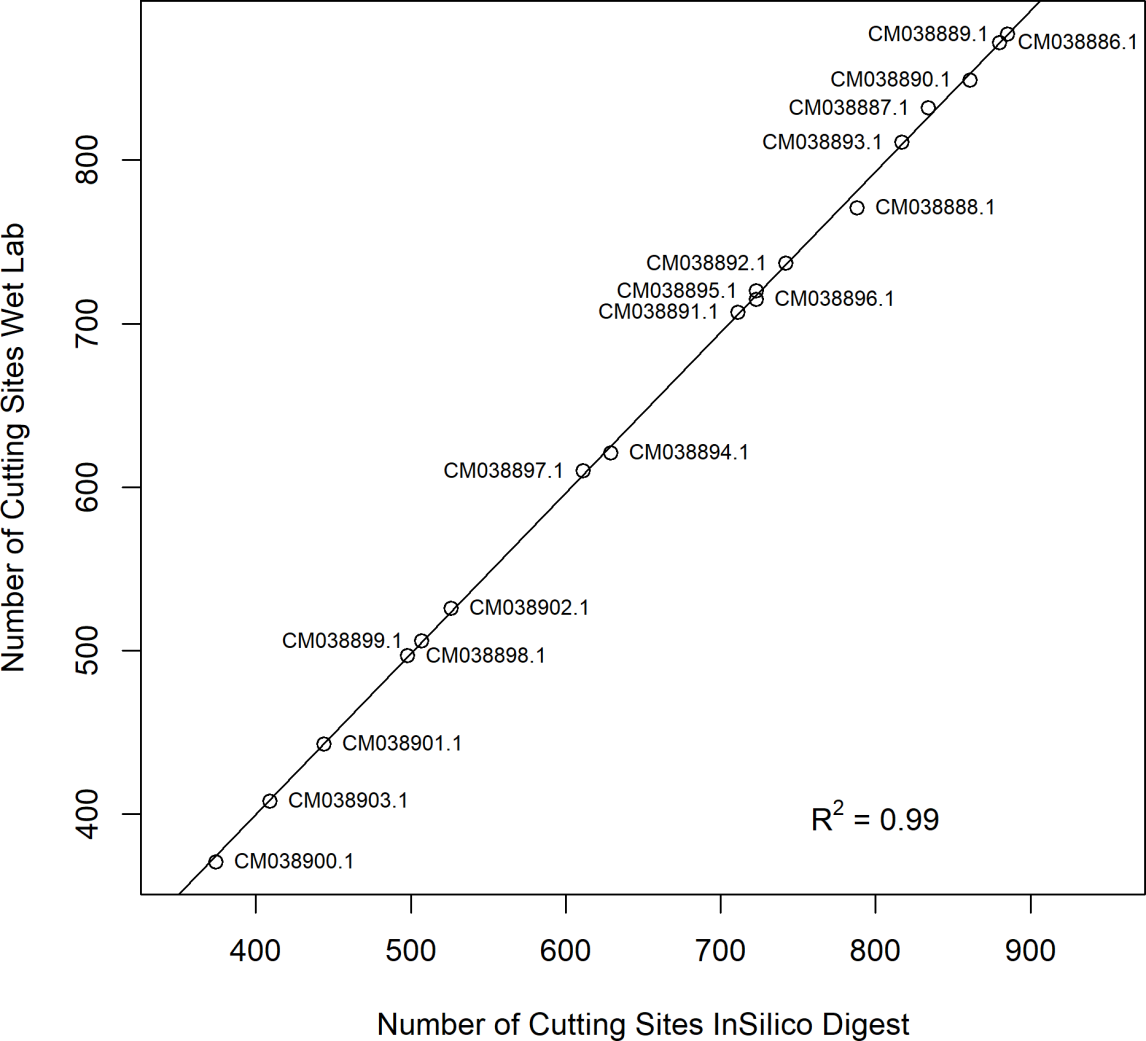
Regression analysis of the number of cleavage sites in the *Pst* reference genome Pst134E16_v1_alt. The *in silico* predicted number of cleavage sites (fragment sizes ranging from 150 to 600 bp) is plotted against the actual cleavage sites across all sequenced isolates (*n* = 152).

An *in silico* restriction digest was performed to assess the coverage and distribution of cleavage sites across the yellow rust reference genome. This analysis included the training set (*n* = 122) and the test set containing UK samples (*n* = 30). Based on the Illumina short-read sequencing approach, only fragments ranging from 150 to 600 bp in length and containing cleavage sites for both restriction enzymes were considered (Table S9). Cleavage sites were merged across all 152 sequenced isolates, with the results shown in Fig 1. The double digest with *MspI* and *PstI* produced cleavage site distributions that closely matched those with the *in silico* analysis, with only minor deviations (~ 0.01%).

### Marker development

For marker development, the training set comprising isolates from Germany and France (*n* = 122 isolates) was used. Group comparisons in the case-control study, along with Fisher’s exact test, revealed no significant InDels, but 14 SNPs showed significant differentiation (*p* < 0.05) and were deemed suitable for distinguishing between the ‘Amboise’, ‘Benchmark’, and ‘Kalmar’ subraces. SNPs that perfectly corresponded to at least one subrace were prioritized, leading to a preselection of seven markers out of the 14 identified SNPs (Table 2, with not selected markers listed in Table S10). Of these, four markers – Marker_5038, Marker_6875, Marker_8441, and Marker_11353 – were successfully established in the laboratory and tested against two independent control groups of yellow rust isolates with known ‘Amboise’, ‘Benchmark’ or ‘Kalmar’ phenotypes.

**Table 2:** Selected markers for differentiation of ‘Amboise’, ‘Benchmark’ and ‘Kalmar’ subraces based on the case-control-study. The number of isolates carrying the reference allele in a homozygous state (0/0), or the alternative allele in a homozygous (1/1), heterozygous (0/1) or not covered (./.) state is listed. The Dataset column indicate, whether the markers were present in differently filtered Datasets 1-3.

Marker	Chr - Pos	Dataset	Race	./.	0/0	0/1	1/1
697	CM038886.1_	1,2,3	‘Amboise’	5	41	3	0
	5027579		‘Benchmark’	11	6	8	0
			‘Kalmar’	0	37	0	0
5038	CM038892.1_	1,2	‘Amboise’	16	7	26	0
	1111760		‘Benchmark’	7	17	1	0
			‘Kalmar’	8	0	29	0
6212	CM038893.1_	1,2	‘Amboise’	14	8	27	0
	4185582		‘Benchmark’	8	16	1	0
			‘Kalmar’	8	0	29	0
6363	CM038893.1_	1,2	‘Amboise’	0	49	0	0
	5274198		‘Benchmark’	1	24	0	0
			‘Kalmar’	3	16	18	0
6875	CM038894.1_	1,2,3	‘Amboise’	0	49	0	0
	2429997		‘Benchmark’	0	25	0	0
			‘Kalmar’	2	16	19	0
8441	CM038896.1_	1,2,3	‘Amboise’	0	49	0	0
	3410845		‘Benchmark’	0	25	0	0
			‘Kalmar’	3	16	18	0
11353	CM038902.1_	1,2	‘Amboise’	10	13	26	0
	1332865		‘Benchmark’	5	20	0	0
			‘Kalmar’	4	22	11	0

For the German control group of yellow rust isolates sampled in 2022 (testG, *n* = 40), marker-assisted analysis and bioassay-based pathotyping, following GRRC standards, yielded consistent assignments for the ‘Amboise’ and ‘Benchmark’ subraces. However, marker analysis failed to accurately predict ‘Kalmar’ subrace isolates (Table S11). For the second control group consisting of UK isolates (*n* = 30), GRRC pathotyping results were available for four isolates, all of which matched the marker-based subrace predictions. For the remaining isolates, a non-GRRC-based pathotyping approach was applied. In this case, nine isolates were correctly assigned based on marker predictions, six were misassigned, and 11 could not be clearly classified (Table S11).

### Genetic distance

**Fig 2:**
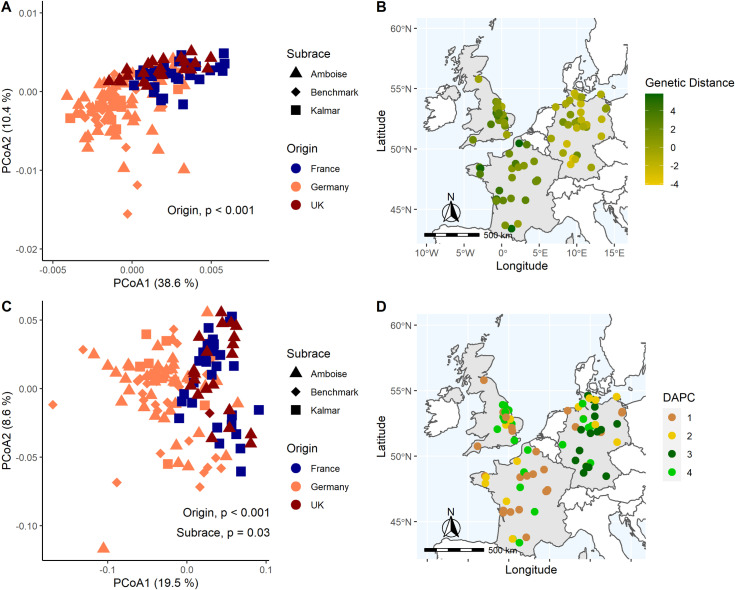
Pairwise genetic distances (Roger’s Distance) analysis. Depending on the filtering steps applied during downstream analysis, Principal Coordinate Analysis (PCoA) and PERMANOVA indicate an effect of isolate origin in A) Dataset 2 (3.464 SNPs) with a more detailed resolution of the genetic relatedness across Europe in B), and the effects of isolate origin and subrace identity in C) Dataset 3 (263 SNPs). Discriminant analysis of principal components (DAPC) clusters were calculated based on the genetic data of Dataset 3 and are presented in D) across France, Germany and the UK. Maps were created using open-source software R with the rnaturalearth (v0.1.0), rnaturalearthdata (v0.1.0) and sf (v1.0-8) packages.

An assessment of genetic distances based on Roger’s Distance (RD) across all sequenced isolates, including the training set and the second test set of UK isolates (*n* = 152), revealed that genetic variation was significantly influenced by the geographical origin of the samples, specifically France, Germany or the UK (PERMANOVA, Figs 2A, 2B and 2C). A gradient in genetic distances was observed from eastern Germany to western France (Fig 2B). Applying a less stringent filter increased the proportion of genetic variance explained - 49% for Dataset 2 (Fig 2A) and 28% for Dataset 3 (Fig 2C) - suggesting that overly strict filtering may have led to a loss of some information (Table S12). However, a subrace-specific effect was detected when SNPs with a minor allele frequency (MAF) < 0.05 and heterozygosity > 0.5 were filtered out, as in Dataset 3 (Fig 2C). To determine whether these significant effects aligned with pathotype characteristics, or more precisely, whether genetic data alone could predict subraces, Dataset 3 was used for artificial clustering via discriminant analysis of principal components (DAPC). The resulting clusters did not correspond to the expected yellow rust isolate pathotypes (Table 3). Instead of three distinct clusters representing the ‘Amboise’, ‘Benchmark’ and ‘Kalmar’ subraces, the algorithm identified four clusters, with all subraces present in each (Table 3). While clusters C1, C3 and C4 were found in France, Germany and the UK, cluster C2 was unique to Germany (Fig 2D). Despite this uniqueness, no significant SNPs distinguished C2 isolates from the others in the case-control-study.

**Table 3:** Discriminant analysis of principal components (DAPC). DAPC clustering is based on genetic data from Dataset 3 (263 SNPs). According to the algorithm, yellow rust isolates were grouped into four clusters (C1 to C4). The cluster assignments of the isolates were then compared to pathotyping results for ‘Amboise’, ‘Benchmark’ and ‘Kalmar’.

Cluster	C1	C2	C3	C4
‘Amboise’	23	28	9	10
‘Benchmark’	1	8	9	8
‘Kalmar’	8	6	17	6

### Genotyping-by-sequencing (GBS) vs whole genome sequencing (WGS)

#### Marker detection.

Following the same marker detection approach applied for GBS, no significant SNPs were identified in the WGS data. However, when combining GBS and WGS data, three additional SNPs were found to be significant, though they did not perfectly correspond to subrace phenotypes (Table S13).

#### Coverage.

**Fig 3:**
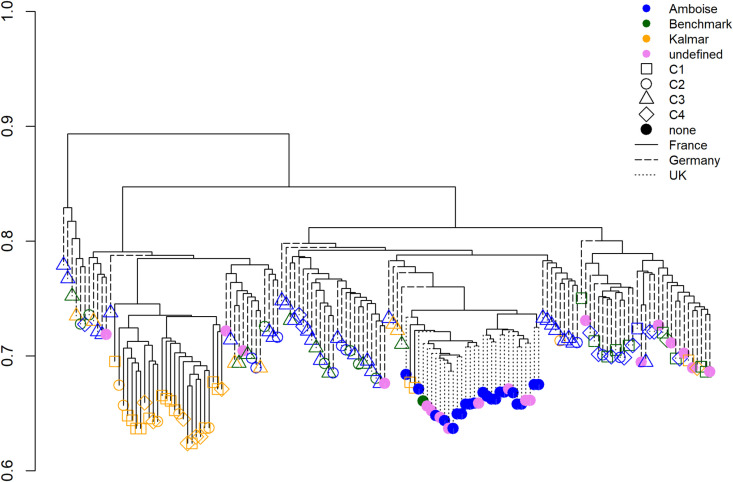
Dendrogram representing differences in GBS coverage among individual isolates. Subraces and isolate origins are indicated by different colors and branch lines, respectively. Point shapes correspond to clusters (C1-C4) generated using discriminant analysis of principal components (DAPC) based on SNP data.

When calculating genome coverage across all isolates, WGS covered 97% of the genome, while GBS covered only 37%. Coverages for genomes of individual isolates ranged from 90 to 98% for WGS but was significantly lower for GBS, ranging from 2.83 to 7.56%, indicating isolate- or group-specific differences. Genetic distances were primarily influenced by the geographic origin of the samples, with the highest combined genome coverage observed in German isolates (29%, *n* = 94), followed by French isolates (25%, *n* = 28 isolates) and British isolates (15%, *n* = 30) (Fig 3).

## Discussion

The differentiation of rust fungi at the species and race levels is commonly achieved through nuclear ribosomal internal transcribed spacer (ITS) region sequencing [43] and genotyping using simple sequence repeats (SSR) markers [14], respectively. However, further classification at the subrace level, such as distinguishing ‘Amboise’, ‘Benchmark’ and ‘Kalmar’ within the yellow rust genetic group PstS10 (also known as ‘Warrior(-)’), requires laborious bioassays on wheat differential sets, as no comparable high-throughput analysis methods are currently available. To facilitate subrace identification, we aimed to develop a marker-assisted approach using genotyping-by-sequencing (GBS). We obtained high-quality GBS data from isolates collected between 2017 and 2021. Based on this training set, we successfully generated functional markers for the two dominant subraces ‘Amboise’ and ‘Benchmark’, and validated these markers using a German test set of isolates collected in 2022.

GBS is a widely used method for SNP detection and molecular marker development. Although it does not provide full genome coverage - partly due to the use of methylation sensitive restriction enzymes - it remains a cost-effective alternative to whole-genome sequencing (WGS) for analyzing population structures and interspecies differences [44]. In this study, we extended this approach to differentiate between subraces with low genetic distances. Successful GBS relies on two key prerequisites: the availability of a high-quality, well-assembled reference genome and the selection of appropriate restriction enzymes.

In 2022, Schwessinger et al. [45] published a chromosome-scale assembly of an Australian yellow rust isolate belonging to the genetic group PstS1 (Pst134E16_v1_alt). Compared to PstS1, the European PstS10 lineage exhibits greater aggressiveness, with additional virulence against *Yr1*, *Yr3*, *Yr4*, *Yr17*, *Yr32* and *YrSp*, but no virulence against *Yr8* (RustWatch, [18]). Molecular studies investigating the genetic mechanisms underlying the gain and loss of virulence or avirulence in different rust pathotypes remain scarce. Virulence shifts may result from point mutations or insertions/deletions (InDels). For instance, Gangwar et al. [46] analyzed 11 Indian yellow rust pathotypes using 24 polymorphic SSR markers and identified 69 alleles, supporting the hypothesis that point mutations play a dominant role. Other studies have similarly suggested that point mutations, rather than InDels, primarily drive virulence changes [12]. However, in the case of the Ug99 lineage of wheat stem rust, *Puccinia graminis* f. sp. *tritici* (*Pgt*), a large 57 kbp insertion in the *AvrSr35* gene was identified as a key factor in virulence evolution [47]. Despite potential structural genome differences between PstS1 and PstS10, including the possibility of larger InDels, and the absence of a high-quality genome assembly for the European PstS10 lineage, all analyses in this study were conducted using the PstS1 reference genome.

The selection of restriction enzymes can be optimized using *in silico* analyses to estimate the number and distribution of cleavage sites across the fungal genome. Several tools are available for this purpose, including CLC workbench (QIAGEN, Aarhus, Denmark) and REDigest for Python [48]. However, many of these tools have limitations, such as licensing restrictions or reduced flexibility. To overcome these constraints, we refined an existing open-source R script, ‘SimRad’ [25], to optimize restriction enzyme selection. After testing various combinations of single and double digests, we selected a double digest with *Msp*I and *Pst*I [28], which provided broad coverage of cleavage sites across the genome. A comparison of *in silico* predicted cleavage sites with experimentally obtained wet-laboratory fragments showed a strong concordance, with a 99.99% overlap across all isolates (Fig 1). Similar results have been reported by Vendrami et al. [49], further highlighting the advantages of *in silico* analysis as a preparatory step for GBS approaches.

A key challenge in GBS data analysis is defining appropriate filtering parameters for variant calling, which strongly depend on the studied organism [50–51]. Applying different thresholds the filter steps significantly affected the number of retained SNPs: the initial dataset (Dataset 0) contained over 12,000 variants with no filters for minor allele frequency (MAF) or heterozygosity (HET). This number dropped to 3,463 in Dataset 2 (MAF > 0.05%, HET = 100%) and further to just 263 SNPs in Dataset 3 (MAF > 0.05%, HET < 50%). Notably, the final filtering step had the most substantial impact, as it revealed a significant subrace-specific effect that was not evident in the less-stringently filtered datasets. Despite this, cluster analyses of all datasets failed to align with known pathotype classifications. Instead of the expected three clusters corresponding to the fungal subraces, DAPC identified four clusters. This inconsistency may be attributed to the highly heterozygous nature of the *Pst* genome [9,52,53], which consists of two haploid nuclei per asexual urediniospore [54]. A recent study of *Puccinia triticina* (*Pt*) demonstrated that a limited number of haplotypes pair freely, driving genetic diversity and the emergence of new strains [55]. Consequently, overly strict heterozygosity filters can lead to substantial information loss. In this study, we developed four KASP markers based on the full dataset from the training set (n > 13,000 variants). Checking the filtered datasets for these markers revealed that applying the stringent filtering criteria of Dataset 3 would have resulted in the loss of two markers. This highlights the importance of carefully balancing filter thresholds to optimize meaningful variant detection while minimizing noise.

When testing the four markers against a German reference set from 2022, results aligned with the phenotypes of ‘Amboise’ and ‘Benchmark’ subrace isolates but failed for ‘Kalmar’. In this case, ‘Kalmar’ isolates were misclassified as either ‘Amboise’ or ‘Benchmark’. This is particularly noteworthy, as ‘Amboise’ combines the virulence properties of both ‘Benchmark’ and ‘Kalmar’ (Table S2), suggesting a greater challenge in distinguishing ‘Amboise’ isolates. Since their establishment in 2017, subraces have remained genetically closely related (RD ranging from 0 to 0.31) and still belong to the same genetic group. This may explain the observed allele distribution among subraces for significant markers (Table 2). In most cases, isolates exhibited either homozygosity for the reference genome or heterozygosity, while homozygosity for the alternate allele was rare. Although not fully resolved in this study, this pattern could be attributed to ongoing evolutionary processes and incomplete differentiation. Isolates likely still predominantly carry the dominant allele but are gradually shifting towards the alternate allele, contributing to location-dependent phenotypic variation. Further supporting this dynamic is the presence of a distinct cluster (DAPC, Cluster 2) found exclusively in Germany (Fig 3C), where all three subraces were represented This suggests that factors beyond genetic divergence, such as environmental conditions, may be shaping genetic diversity and influencing phenotypic variation within these populations. Consistent with this, recent monitoring by the RustWatch project has identified a trend in Germany, where ‘Amboise’ and ‘Benchmark’ are becoming dominant over ‘Kalmar’ isolates.

Finally, we evaluated marker functionality in a control group by predicting the phenotypes of 30 UK isolates collected in 2021 based on genetic markers detected in GBS data. However, the classification system for yellow rust races in the UK differs substantially from that of GRRC, and phenotyping only partially matched our predictions. Interestingly, genetic distance analysis revealed a significant origin effect, which was also reflected in genome coverage differences. The combined GBS-generated sequences covered only 15% of the fungal genome in UK isolates, compared to 25% for French isolates (*n* = 28) and 29% for German isolates (*n* = 94). Given that the UK and French samples had even higher total read counts and mapping percentages (Fig S2), these coverage differences may be attributed to epigenetic factors, specifically DNA methylation. Methylation-sensitive restriction enzymes such as *Msp*I, used in the GBS protocol, cannot cleave methylated DNA, potentially reducing genome coverage without altering the actual sequence [56]. Methylation plays a key role in adaptation, as observed in plants responding to biotic and abiotic stresses [57], bacterial pathogenicity regulation [58], and fungal biological functions [59]. The lower genome coverage of UK isolates, along with a reduced number of cleavage sites (Table S9), suggests an additional origin-driven divergence within the ‘Warrior(-)’ race group, influenced by distinct environmental conditions in the UK compared to continental Europe. These findings align with a study by de Vallavieille-Pope et al. [60], which demonstrated that ‘Warrior(-)’ isolates exhibit different responses to climatic conditions. Furthermore, a more recent study highlighted the adaptability of ‘Warrior(-)’ isolates to varying regional climates in France, facilitating the replacement of previous populations [61]. Similarly, shifts in yellow rust populations between northern and southern France [62] warrant further investigation of environmental factors in shaping genetic diversity.

## Conclusion

Using GBS, we established four KASP markers capable of distinguishing between the ‘Amboise’ and ‘Benchmark’ subraces within the PstS10 genetic group. This provides a valuable tool for virulence monitoring initiatives such as the Global Rust Reference Center (GRRC, Rustwatch [18]) to track further developments in yellow rust populations. However, for improved resolution of subrace-specific properties, future studies should prioritize long-read sequencing approaches and subrace-specific genome assemblies.

Although not the primary focus, this study also revealed a strong effect of isolate origin on GBS data, particularly when comparing samples from France, Germany and the UK. Given that sexual reproduction of yellow rust is rare in Europe, asexual recombination events are likely the main drivers of diversity under changing environmental conditions. Nonetheless, future research should consider both reproductive modes to better understand genotype-environment interactions shaping and shifting yellow rust population structures in Europe.

## Supporting information

S1 Fig Galaxy workflow illustrating the processing of GBS sequences from raw data to the final table containing quality-filtered variants. (TIF)

S2 Fig Unmapped vs. mapped reads. Variance in A) the total number of reads and B) the percentage of mapped reads from the GBS approach, summarized for isolates originating from France, Germany and the UK.
(TIF)

S1 File Modified SimRad R Script for In Silico analysis of restriction enzymes cleavage sites. (TXT)

S2 File File contains Tables S1 to S13. (XLSX)
