# Supplementary figures and images for: Differentiation of European yellow rust subraces within the ‘Warrior(-)’ genetic group
Source: PLoS One. 2025 May 23;20(5):e0323046. doi: 10.1371/journal.pone.0323046 (PMC12101709; doi:10.1371/journal.pone.0323046)

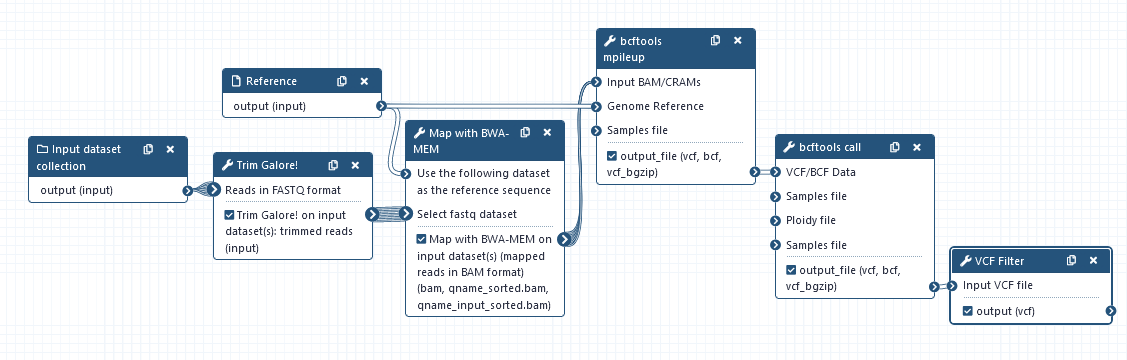

Supplement: S1 Fig — (TIF) [file pone.0323046.s001.tif]

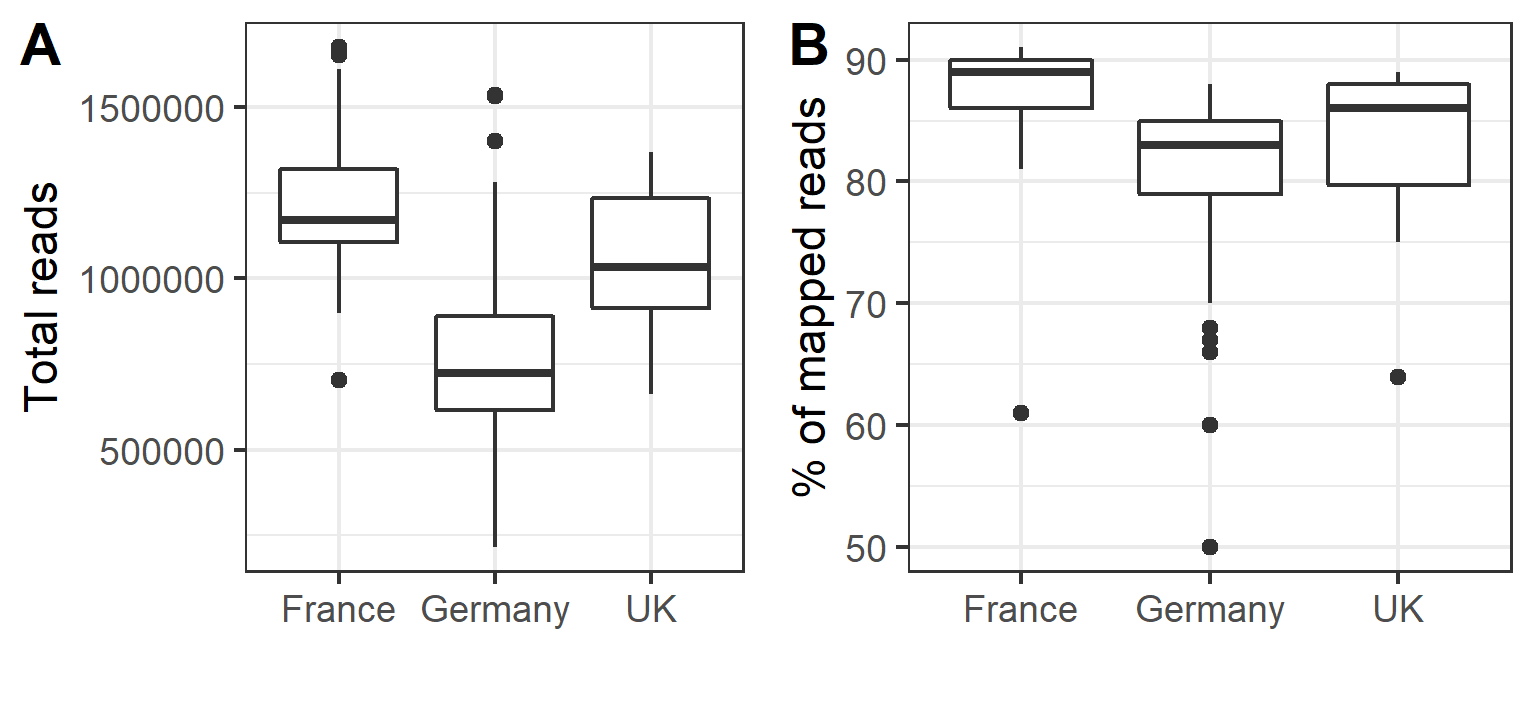

Supplement: S2 Fig — Variance in A) the total number of reads and B) the percentage of mapped reads from the GBS approach, summarized for isolates originating from France, Germany and the UK. (TIF) [file pone.0323046.s002.tif]
